# Antiphospholipid syndrome with major arterial thrombosis, presenting as pulmonary thromboembolism, cerebrovascular accident, and coronary artery disease: A case report and literature review

**DOI:** 10.1002/ccr3.9254

**Published:** 2024-08-05

**Authors:** Aria Shirani, Morteza Daraei, Aryan Shirani

**Affiliations:** ^1^ Research Scholar, School of Medicine Iran University of Medical Sciences Tehran Iran; ^2^ Assistant Professor of Internal Medicine, Department of Internal Medicine Imam Khomeini Hospital Complex Tehran Iran

**Keywords:** antiphospholipid syndrome, arterial thrombosis, case report: APS, PTE, pulmonary embolism

## Abstract

Antiphospholipid syndrome is an immunopathologic disorder that should be considered in all patients with recurrent and/or unexplained thromboembolic events. Antiphospholipid antibodies are diagnostic markers, and anticoagulation therapy is the therapeutic and preventive strategy. Long‐term anticoagulation therapy is necessary, with careful attention to potential bleeding complications.

## 1 INTRODUCTION

Antiphospholipid syndrome (APS) is an autoimmune hypercoagulable state where autoantibodies directed against phospholipids or their bindings, including β2‐glycoprotein‐1 and prothrombin, cause predisposition to arterial or venous thrombosis along with pregnancy events.^1^ It is more commonly observed in women than men, and the average age of onset is around 50. In the US, the prevalence of APS is 5 per 10,000, and the annual incidence rate is approximately 2.1 per 100,000.^2^ Involved patients have a thrombophilic condition, which may lead to thrombosis in major or minor vessels and also obstetrics adverse outcomes, including abortion and preeclampsia.^3^ Although the most common presentation is deep venous thrombosis, arterial thrombosis is more critical and likely deadly. Stroke is the most prevalent arterial event in APS, while coronary involvement is rare (20% vs. 2%).^4^ The main diagnostic criteria of APS include at least one clinical criterion plus a laboratory‐based criterion.

Clinical criteria include:

1. Vascular thrombosis, including arterial, venous, and minor vessel thrombosis episodes.
2. Obstetrics morbidity, including at least one stillbirth after 10 weeks gestation in a morphologically normal fetus, or at least one premature birth before 34 weeks gestation due to eclampsia, severe preeclampsia, or placental insufficiency in a morphologically normal fetus.
3. At least three unexplained sequential spontaneous abortions before 10 weeks gestation in the absence of maternal anatomic, hormonal, and chromosomal abnormalities.

Laboratory criteria consist of:

1. Presence of medium or high titers of lupus anticoagulant (LA), anti‐beta‐2 glycoprotein‐1 (Anti‐β2GP1) antibodies, and/or anti‐cardiolipin (aCL) antibodies on at least two occasions with a time interval of at least 12 weeks.^5^

Despite many other forms of autoimmune disorders, APS cannot be managed by immunomodulating therapies. So, the primary therapeutic approach in nonpregnant adults is anticoagulation with vitamin K antagonists to achieve the targeted international normalized ratio (INR). However, this strategy is not always feasible, especially when the risk of bleeding is high.^6^ In this case report, we present a patient with APS who experienced three major arterial thromboses, including pulmonary, cerebral, and coronary arteries. The patient had to undergo an emergent craniectomy due to increased intracranial pressure (ICP), which carries a potential risk for intracranial hemorrhage due to the anticoagulation required for thrombosis treatment. Each event carries a high mortality risk, and managing such a complicated situation is a great challenge.

## 2 PATIENT INFORMATION

A 33‐year‐old man with a recent history of a cerebrovascular attack (CVA) presented to the emergency department due to a sudden onset of dyspnea. The patient reported that the dyspnea started a day before while he was resting in bed. It was so severe that he could not even walk to the bathroom. Additionally, he experienced pleuritic chest pain in the right hemithorax that developed along with dyspnea.

The patient experienced a CVA 3 weeks ago in his medical history. According to his medical documents, he visited the neurologic emergency after a sudden onset of aphasia and right hemiparesis. He was given medical therapy to manage left middle cerebral artery (MCA) thrombosis. After a few days of admission, he developed symptoms and signs of ICP and underwent an emergent craniectomy to reduce the ICP. In addition, 2 years ago, the patient experienced another thrombotic event when he was diagnosed with acute coronary syndrome (ACS) due to left anterior descending (LAD) artery occlusion. After that, the patient underwent coronary angioplasty and received ischemic heart disease (IHD) medical therapy to prevent heart failure.

There is a significant family history of IHD in his father and uncle, who were diagnosed with the condition at the ages of 60 and 54, respectively. The patient mentioned that he used tobacco occasionally before experiencing these health issues.

## 3 CLINICAL FINDINGS

At the presentation, the patient was awake and alert but had tachypnea and tachycardia with rates of 26/min and 124/min, respectively. Otherwise, the hemodynamic state was stable, and fever was not detected. His graft for recent craniectomy was apparent from his scalp. The cardiac auscultation revealed no abnormal sound; the lungs were bilaterally expansile, and no abnormal sound was heard. The forces of the left limbs were reduced compared to those of the right limbs. The remaining examination revealed no significant abnormality.

## 4 DIAGNOSTIC ASSESSMENT

**FIGURE 1:**
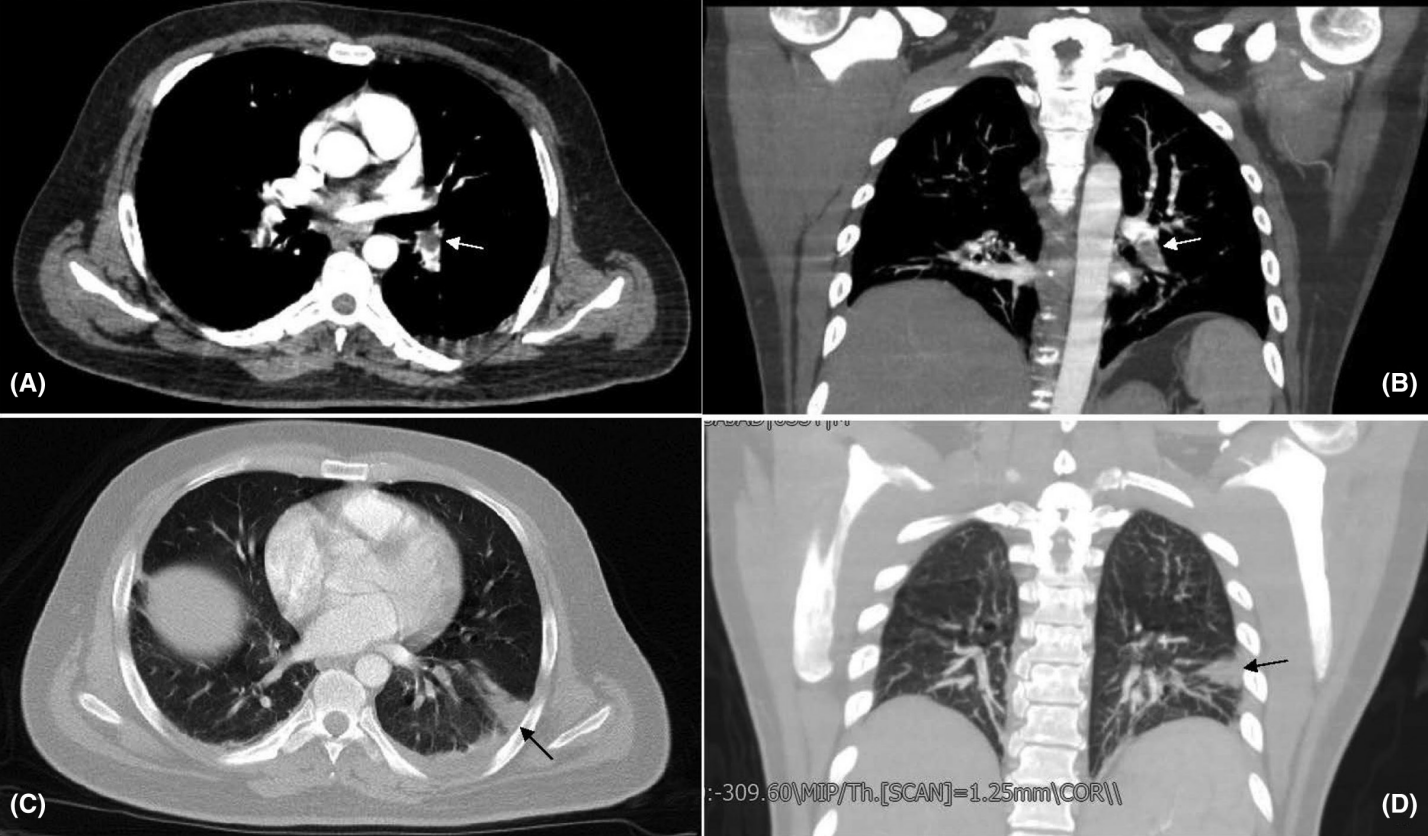
CT pulmonary angiogram (CTPA) revealed a filling defect at the bifurcation of the left pulmonary artery extending to the left lower lobe artery, consistent with arterial embolism (A and B, white arrows). A focus of wedge‐shaped infarction is evident at the lateral basal segment of the left lower lobe (C and D, black arrows).

**FIGURE 2:**
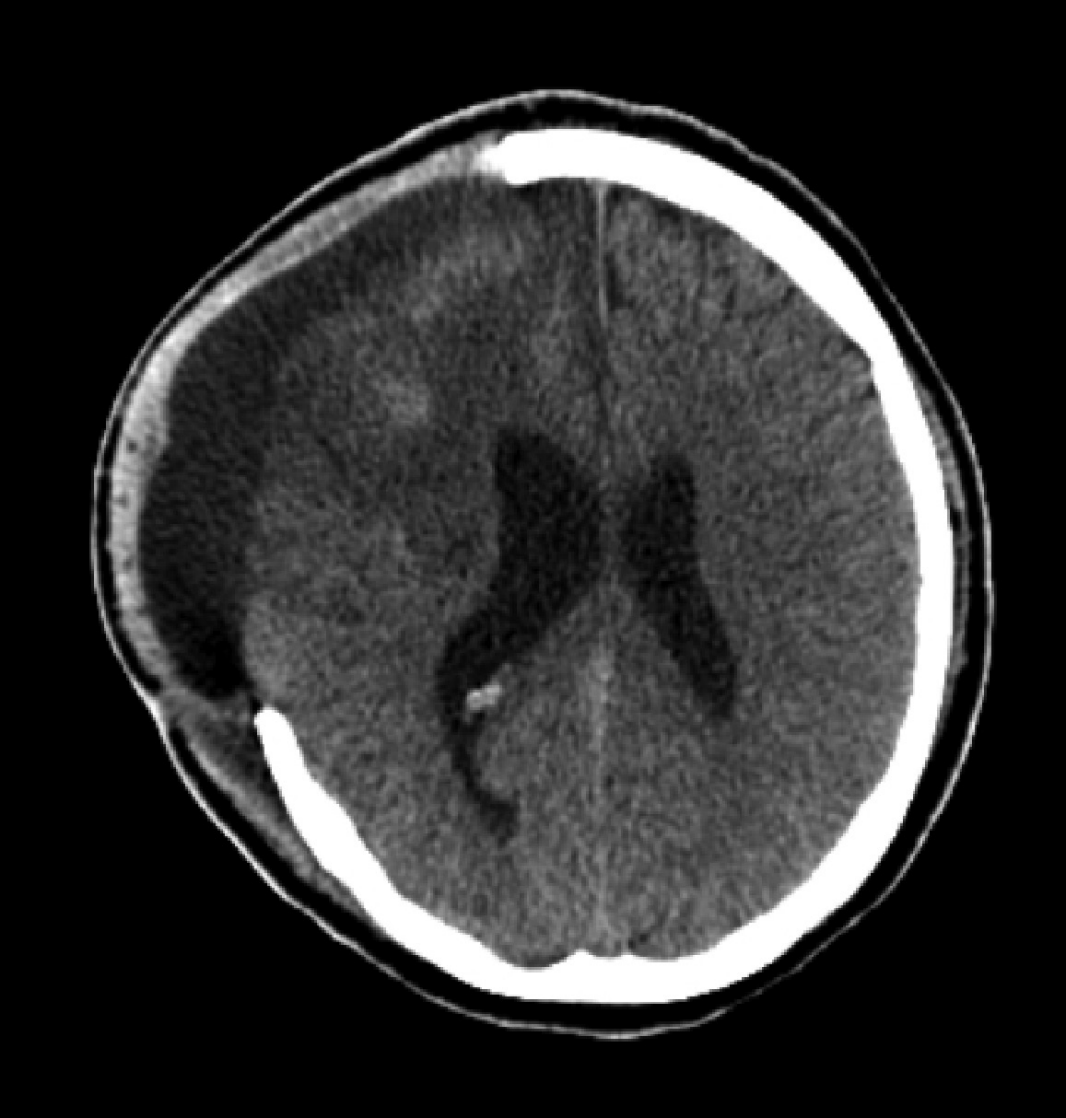
Brain CT scan of the patient shows hypodensities in the right frontal, temporal, and parietal lobes and evidence of craniectomy due to a recent CVA. There was no sign of bleeding, permitting judicious anticoagulation therapy.

Initial laboratory testing, which included complete blood count, liver function test, renal function test, urine analysis, and electrolytes, were unremarkable. Inflammatory markers, including C‐reactive protein, were negative, making underlying infection unlikely. Lipid profile did not show signs of dyslipidemic disorders, with cholesterol and triglyceride levels within the normal range. Due to high clinical suspicion of pulmonary thromboembolism (PTE), a pulmonary computed tomography angiography (CTA) was performed. This modality revealed filling defects in segmental arteries of the left lower lobe (LLL) and right lower lobe (RLL) of the lungs. In addition, a wedge‐shaped focus of infarction was seen in the basolateral segment of LLL (Figure 1). These findings were consistent with PTE. Following this diagnosis, we started anticoagulation therapy with the infusion of unfractionated heparin (UFH), with monitoring of PTT. Before initiating intravenous anticoagulation therapy, we performed a brain CT scan based on the patient's risk of bleeding due to a recent craniectomy, which showed no hemorrhage, and the only finding was evidence of a recent craniectomy (Figure 2). According to the suspicious history of the patient, we requested serologic tests for APS, including LA, anti‐β2GP1 IgG, and aCL IgG. The results were positive, with anti‐β2GP1 and aCL titers of 25.6 (negative≤8) and 35.6 (negative≤11), respectively. Additionally, anti‐nuclear antibody was weakly positive, and anti‐double stranded DNA (anti‐dsDNA) titer was three times above the upper limit of normal. Meanwhile, serum complement levels were within the normal range. Moreover, testing for other thrombophilic disorders, including factor V Leiden and prothrombin G20210A mutation, was negative.

## 5 MANAGEMENT

Based on clinical suspicion of PTE, after taking neurosurgical permission for anticoagulation therapy, infusion of UFH was started with the monitoring of PTT every 6 hours. Supportive care, including IV hydration and electrolyte balancing, was part of our initial treatment. The patients also received gastrointestinal bleeding prophylaxis with IV pantoprazole.

## 6 FOLLOW‐UP AND OUTCOME

After several days of the mentioned therapies, we observed clinical improvement. Following bridge therapy for warfarin, the patient was discharged with warfarin and aspirin and scheduled for weekly outpatient follow‐up visits to monitor the INR. Three months later, we rechecked the LA and titers of anti‐β2GP1 and aCL. All these values tested positive, confirming the APS diagnosis. During these 3 months, the patient did not experience any thromboembolic events or bleeding issues.

## 7 DISCUSSION

APS, an antibody‐mediated acquired thrombophilia, is characterized by recurrent arterial and venous thrombosis and pregnancy complications, including pregnancy loss. When APS occurs alongside other autoimmune diseases, mainly systemic lupus erythematosus (SLE), it is considered secondary APS. On the other hand, primary APS refers to APS occurring alone in a patient. The diagnosis of APS should be considered when a thromboembolic event occurs in a young patient without risk factors.^7^

APS is a significant cause of pregnancy loss, stroke, DVT, and MI in the United States.^8^ Although APS poses patients to thromboembolic events, there are few case reports that introduce a patient with three major thromboembolic events, including MI, stroke, and PTE.^9^, ^10^ While most of the previous case reports suggest arterial and venous thrombosis in maximal two organs, our case was a rare instance of APS involving three critical body sites, which has rarely been reported.^9^, ^11^

The precise pathophysiological mechanism of the hypercoagulable state in APS still needs to be fully understood. However, it appears to be caused by direct injury to the vascular endothelium. This injury exposes phospholipids to plasma proteins such as β2GP1 and prothrombin, forming neoantigens that activate the immune system. This activation represents the first step in inflammation and thrombus formation.^7^ Recently, it has been hypothesized that phagocytic system dysfunction, complement activation, and imbalance in type 1 and type 3 interferons play a remarkable role in APS‐related obstetric complications.^1^, ^12^

The differential diagnosis of APS includes acquired and congenital causes of thrombophilia. Heritable causes may result in arterial, venous, or arterial and venous thrombosis. When an unexplained thrombosis occurs in a patient younger than 55, it is crucial to rule out congenital thrombophilia. The acquired causes of thrombophilia consist of a wide range of risk factors that are sometimes feasible to understand. For instance, a history of major abdominal surgery or trauma serves as an illustrative example. On the other hand, diagnosis of polycythemia vera and myeloproliferative disorders should prompt the clinician assessment based on the hematological and other organ‐involving abnormalities. Furthermore, precise physical examination and history taking with an emphasis on major abdominal surgery, obesity, and history of recent trauma or oral contraceptive usage are necessary. Precise lab tests to exclude other causes of thrombophilia may extend to complete blood count, renal function test, and urine analysis to rule out nephrotic syndromes, liver function tests, factor V Leiden, prothrombin G20210A mutation and ANA.^13^, ^14^, ^15^

The treatment of our patient was a clinical challenge. History of the previous craniectomy 2 weeks ago due to a massive ischemic stroke predisposes him to intracranial bleeding from anticoagulant therapy. For the acute treatment of PTE, we used UFH because of the lower half‐life and availability of an antidote, protamine sulfate, compared to low molecular weight heparins. After treatment with UFH and resolving symptoms, oral anticoagulation should be prescribed for long‐term use. The privileged choice of anticoagulation therapy is warfarin, a vitamin K antagonist.^16^ With the widespread use of direct oral anticoagulants (DOACs) like apixaban in recent years, multiple studies compare the effectiveness and adverse outcomes of these drugs. Compared to warfarin, apixaban shows a lower risk for major bleeding events in the general population, especially in patients with atrial fibrillation.^17^ Meanwhile, multiple studies reveal higher efficacy of oral warfarin with tight INR in preventing recurrent thromboembolic events and lower risk for major bleeding events in comparison to DOACs like apixaban in patients with APS.^13^, ^16^ In a systematic review and meta‐analyses in 2022, recurrent thrombotic events were 8.8% in the apixaban group and 3.1% in the warfarin group, with no difference in all‐cause mortality. Conversely, patients' compliance to control INR is a negative point for warfarin, while apixaban requires no dose adjustment.^18^ Because of recurrent arterial events, low‐dose aspirin was considered together with warfarin, with targeted INR between 2 and 3.^19^ Noteworthy, the patient neither experienced hemorrhagic diathesis nor thromboembolic event in 3 months of follow‐up.

Our patient also had positive ANA and significantly elevated anti‐dsDNA titer as well as antiphospholipid antibodies. Meanwhile, other manifestations of SLE (both clinical and laboratory) were not present. As a result, based on EULAR/ACR classification criteria, the patient was not classified as having SLE.^20^ However, due to the high specificity of significant elevation of anti‐dsDNA for SLE, the patient should be closely followed up for SLE symptoms and its major organ complications.^21^ We used the Global Antiphospholipid Syndrome Score (GAPSS) to estimate the likelihood of recurrent thrombotic events in the context of SLE. In this case, the score of 13 (with a threshold of 6) indicated a high risk of recurrent thrombosis, necessitating strict adherence to the anticoagulation regimen.^22^

## 8 CONCLUSION

Concurrent presentation of three major thromboembolic events, including PTE, ischemic stroke and coronary artery disease, is a rare presentation of APS. The diagnosis of APS is based on thrombotic or obstetrics complications and serologic studies. Patients with APS are susceptible to recurrent thromboembolic events, and long‐term anticoagulation is required. The APS should be considered in all patients who present to clinics with an unexplained thromboembolic event, especially in a setting like the aforementioned young person with episodes of both arterial and venous thrombosis.

## AUTHOR CONTRIBUTIONS

**Aria Shirani:** Conceptualization; data curation; writing – original draft. **Morteza Daraei:** Conceptualization; supervision; validation; writing – review and editing. **Aryan Shirani:** Data curation; project administration; supervision; writing – original draft; writing – review and editing.

## FUNDING INFORMATION

There is no funding to report for this submission.

## CONFLICT OF INTEREST STATEMENT

All authors have declared no conflict of interest.

## ETHICS STATEMENT

The patient gave written informed consent, and the study was conducted per the Helsinki Declaration, revised in 2013. The signed consent form is available as Wiley's consent form.

## CONSENT

Written informed consent was obtained from the patient to publish this case report per the journal's policy.

## Data Availability

All data underlying the results are available as part of the article and no additional source data are required.
